# Effects of High-Definition Anodal Transcranial Direct Current Stimulation Applied Simultaneously to Both Primary Motor Cortices on Bimanual Sensorimotor Performance

**DOI:** 10.3389/fnbeh.2017.00130

**Published:** 2017-07-12

**Authors:** Nils H. Pixa, Fabian Steinberg, Michael Doppelmayr

**Affiliations:** ^1^Institute of Sport Science, Johannes Gutenberg-University Mainz, Germany; ^2^Centre for Cognitive Neuroscience, Paris Lodron-University Salzburg, Austria

**Keywords:** brain stimulation, multichannel tDCS, motor cortex, motor performance, sport stacking, multiple-day application

## Abstract

Many daily activities, such as tying one’s shoe laces, opening a jar of jam or performing a free throw in basketball, require the skillful coordinated use of both hands. Even though the non-invasive method of transcranial direct current stimulation (tDCS) has been repeatedly shown to improve unimanual motor performance, little is known about its effects on bimanual motor performance. More knowledge about how tDCS may improve bimanual behavior would be relevant to motor recovery, e.g., in persons with bilateral impairment of hand function. We therefore examined the impact of high-definition anodal tDCS (HD-atDCS) on the performance of a bimanual sequential sensorimotor task. Thirty-two volunteers (age *M* = 24.25; *SD* = 2.75; 14 females) participated in this double-blind study and performed sport stacking in six experimental sessions. In sport stacking, 12 specially designed cups must be stacked (stacked up) and dismantled (stacked down) in predefined patterns as fast as possible. During a pretest, posttest and follow-up test, two sport stacking formations (3-6-3 stack and 1-10-1 stack) were performed. Between the pretest and posttest, all participants were trained in sport stacking with concurrent brain stimulation for three consecutive days. The experimental group (STIM-M1) received HD-atDCS over both primary motor cortices (M1), while the control group received a sham stimulation (SHAM). Three-way analysis of variance (ANOVA) revealed a significant main effect of TIME and a significant interaction of TIME × GROUP. No significant effects were found for GROUP, nor for the three-way interaction of TIME × GROUP × FORMATION. Further two-way ANOVAs showed a significant main effect of TIME and a non-significant main effect for GROUP in both sport stacking formations. A significant interaction between TIME × GROUP was found only for the 3-6-3 formation, indicating superior performance gains for the experimental group (STIM-M1). To account and control for baseline influences on the outcome measurements, ANCOVAs treating pretest scores as covariates revealed a significant effect of the stimulation. From this, we conclude that bilateral HD-atDCS over both M1 improves motor performance in a bimanual sequential sensorimotor task. These results may indicate a beneficial use of tDCS for learning and recovery of bimanual motor skills.

## Introduction

Transcranial direct current stimulation (tDCS) is a non-invasive method for modulating neuronal excitability. It does so by altering the neuronal membrane at rest, and appears to be a promising tool for improving motor skills and initiating neuronal plasticity in the motor cortex (Kidgell et al., 2013), with its strongest effects seen when co-applied with motor activity (Reis and Fritsch, 2011; Saucedo Marquez et al., 2013). In tDCS, a weak and constant current in the range of 0.5–2 milliampere (mA) is applied to the cerebral cortex through the skull (Nitsche and Paulus, 2000; Priori, 2003). This is thought to provoke a polarity-specific subthreshold influence on the resting membrane potential (Nitsche and Paulus, 2000). While anodal stimulation facilitates spontaneous neuronal activity, it is assumed that cathodal tDCS leads to the converse effect and results in inhibition of the stimulated brain area (Stagg and Nitsche, 2011; Dayan et al., 2013). Direct current is conventionally applied using two or more rectangular sponge or rubber electrodes with a size of 25–35 cm^2^ (Ruffini et al., 2014). The so-called high-definition tDCS (HD-tDCS) is a modified tDCS-method that uses smaller (1–5 cm^2^) and usually round sponge or gel electrodes (AgCl) to stimulate the brain region of interest. Based on physiological and computational studies, it is thought that HD-tDCS applies the current flow more focally, allowing a more target-oriented application of transcranial direct currents (Edwards et al., 2013; Miranda, 2013; Villamar et al., 2013; Nikolin et al., 2015; Alam et al., 2016). The first attempts by our group to target the motor domain using this technique have already indicated successful modulation of motor adaptation (Doppelmayr et al., 2016) and sensorimotor performance (Pixa et al., 2017).

There is growing evidence that tDCS of the motor cortex improves motor performance and motor learning in both healthy individuals as well as patients suffering from diverse neurological diseases (Bastani and Jaberzadeh, 2012; Brunoni et al., 2012). Such positive tDCS effects on motor skills have been reported for single-session applications, as well as for multiple applications over several days of training (Vines et al., 2008; Reis et al., 2009, 2015; Schambra et al., 2011; Saucedo Marquez et al., 2013; Waters-Metenier et al., 2014), with prolonged improvement of up to 3 months (Reis et al., 2009), suggesting an impact on neuroplasticity mediated by synaptic long-term potentiation (LTP) within the motor cortex (Liebetanz et al., 2002; Reis and Fritsch, 2011; Stagg and Nitsche, 2011). Unfortunately, most tDCS studies have focused almost exclusively on unimanual motor performance (for an overview, see Buch et al., 2017).

Since humans spend over half of their time each day grasping and manipulating objects with both of their hands (Kilbreath and Heard, 2005), motor behavior in everyday life—whether in sport or in occupational and recreational activities—most often involves skilled and precise bimanual movements. There is also evidence that bimanual motor training leads to improvements in hand and arm function in the paretic limb, e.g., in stroke patients (Cauraugh et al., 2010). Consequently, more research is needed on how to support bimanual training with specific stimulation protocols. tDCS appears to be a promising tool in this regard, due to evidence of its ability to improve unimanual motor skills, its low cost, its relative ease of use and its noninvasive nature.

There is consensus that bimanual movements are not driven by a single brain region; rather, the complex organization of bimanual behavior is based on a motor network within and between both brain hemispheres (Swinnen and Wenderoth, 2004; Grefkes et al., 2008). In this neural network, the primary motor cortices (M1) play an essential role due to their key functions in goal-oriented movement execution (Halsband and Lange, 2006; Hardwick et al., 2013). In addition, M1 is associated with the consolidation of newly learned motor skills (Brashers-Krug et al., 1996; Doyon and Benali, 2005; Dayan and Cohen, 2011; Wessel et al., 2016). Further insights into the cortical activation pattern of bimanual motor behavior come from transcranial magnetic stimulation (TMS) studies. McCombe Waller et al. (2008) demonstrated strong bilateral excitation in left and right M1, when both hands were used simultaneously. Changes in motor cortical excitability due to bimanual training of bilateral wrist movements revealed strong activation of the M1 in both hemispheres (Neva et al., 2012). During synchronous (i.e., in-phase) movements of both wrists, which requires intra- and intercortical interactions, Byblow et al. (2012) reported decreased intracortical inhibition (ICI) as well as interhemispheric inhibition (IHI), with no changes in ICI or IHI during anti-phase movements. In line with that finding are observations of increased bilateral cortical activity in asynchronous compared to synchronous bilateral tasks (Haslinger et al., 2004). Considering these cortical activation patterns during bimanual movements, it seems appropriate to target both M1s as a first step when investigating tDCS effects on bimanual motor performance. This, however, has not been done systematically as of yet. Consequently, reports on the effects of tDCS on bimanual behavior and motor skill learning are scarce. Carter et al. (2015) used tDCS to modulate the excitability of the supplementary motor area (SMA) and to investigate polarity-dependent consequences in bimanual behavior. They reported that only anodal tDCS enhanced the stability and consistency of bimanual anti-phase wrist movements along a delayed transition from anti-phase to in-phase movements (Carter et al., 2015). The same group also showed that anodal tDCS over the SMA facilitates the switch between anti-phase and in-phase when performed intentionally (Carter et al., 2017). Others (Vancleef et al., 2016) applied tDCS to the left M1 (lM1) or left dorsolateral prefrontal cortex (lDLPFC) concurrently over 4 days (one session per day) of learning a complex bimanual task. Bimanual motor skill learning did not differ between the groups, nor were there any neurophysiological changes following tDCS. The lines of research focusing on bimanual tasks have either stimulated the SMA or only one M1. To our knowledge, the extent to which simultaneous stimulation of both M1s impacts bimanual performance has only been investigated by two studies. In the first, Gomes-Osman and Field-Fote (2013) applied anodal tDCS using two anodes, one over the left and one over the right M1, with two bilateral cathodes over the left and right supraorbital cortex. tDCS was applied in between performance of a bimanual typing task for five consecutive days. The authors observed significantly greater task improvements from day 1 to day 5 for the stimulated participants compared to sham-stimulated control group. However, this effect vanished after a retention interval of 1 week, possibly due to the non-concurrent timing of tDCS with motor practice. The second study was recently published by our group and showed improvements in unimanual and bimanual dexterity assessed with the Purdue Pegboard Test (PPT) after HD-atDCS bilateral M1 co-applied with motor training for three consecutive days (Pixa et al., 2017). Here, prolonged improvements were seen after a retention interval of 1 week, indicating increased bimanual motor learning and consolidation.

To summarize, it has been consistently shown that unimanual motor performance can be improved by anodal stimulation of M1, but little is known about the effect on bimanual motor performance. Thus, further exploration of whether tDCS can improve bimanual motor performance is required—a request that has been frequently made, since many daily activities require skillful coordinated use of both hands to interact with the surrounding environment. Such knowledge is relevant beyond a simply theoretical point of view, given that a) rehabilitation of bimanual motor behavior is essential for healthy and independent living in old age or after disease (e.g., for persons with bilateral hand dysfunction) and b) patients benefit from bimanual training, e.g., during recovery after stroke. Therefore, the central purpose of the present study was to investigate the effects of HD-atDCS on performance in a bimanual sequential sensorimotor task. The present approach uses a sport stacking task that requires the subject to build up (stack up) and dismantle (stack down) a formation of objects (cups) in a predefined and fixed formation (objects, actions and order) as quickly and as accurately as possible using both hands. Since sport stacking has already been the object of investigation in earlier studies concerning saccadic eye movements and attention (Foerster et al., 2011, 2012), impact on hand-eye coordination and reaction time (Udermann et al., 2004) and influence on motor and cognitive functions (McKune et al., 2011), it seems perfectly suited to our investigation. In addition to requiring well-established coordination between the upper limbs, sport stacking requires bimanual goal-oriented dexterity to precisely grasp and manipulate the cups at a high speed. Furthermore, it has several advantages, such as novelty, competitive character, task variations, ecological validity and no ceiling effects expected in a short time period (Foerster et al., 2011, 2012).

We hypothesized that sport stacking training conducted over multiple days with co-application of HD-atDCS to both M1s simultaneously would lead to superior performance gains (expressed in shorter movement time) compared to a sham-stimulated control group.

## Materials and Methods

### Participants

An *a priori* sample size calculation (G*Power 3.1.9.2) specified a total sample size of 28 participants to obtain a moderate effect size $\omega_{\text{p}}^{\text{2}}$ = 0.25 and power of 1-*β* = 0.80 with a mixed design and a significance level of *α* < 0.05. In order to prevent loss due to drop out and to keep the number of participants constant, a total of 32 volunteers (14 females) were recruited to participate in the multiple-session double-blind study (age *M* = 24.25; *SD* = 2.75). All participants were right-handed, healthy, without neurological or psychological disorders, and with normal or corrected-to-normal vision. None had previous experience with the sport stacking task. This study was carried out in accordance with the recommendations of the Deutsche Gesellschaft für Psychologie (DGPs) with written informed consent from all subjects. Prior to the experiment, all subjects were informed about all relevant issues of this study and gave written informed consent in accordance with the Declaration of Helsinki. Ethical approval was obtained from the DGPs. All participants were asked to disclose pre-existing neurological and psychological conditions, severe medical conditions and drug intake. Dominant right-handedness was evaluated using the shortened (10-item) Edinburgh Handedness Inventory (Oldfield, 1971). The laterality index (LI) showed an average of 88.38 (*SD* = 18.11), indicating strong right-hand lateralization (max. LI = 100). Fine sensorimotor skills and (bi-)manual dexterity were tested with the PPT (Pegboard Model 32020, Lafayette Instrument Company, Lafayette, IN, USA). Before the first task, the volunteers were randomly assigned to a group that would either receive anodal HD-tDCS over both M1s (STIM-M1, *n* = 16 (8 females)) or a sham stimulation (SHAM, *n* = 16 (6 females)). The participants were asked to report any side effects of the stimulation and none were reported. There were no differences between groups (STIM-M1 vs. SHAM) on self-reported measures of attention, fatigue and discomfort. Neither the LI nor the PPT showed significant differences between the groups (STIM-M1 vs. SHAM) prior to the experiment.

### Experimental Setup

**Figure 1:**
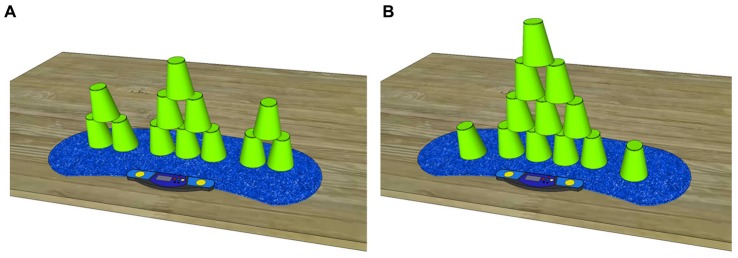
Both required sport stacking formations in an up stacked fashion **(A)** 3-6-3 and **(B)** 1-10-1 stack. Sport stacking must be performed from left to right by stacking up and stacking down the 12 cups in the visualized pattern.

The experiment took place in a quiet room with normal lighting. The official sport stacking equipment according to the World Sport Stacking Association (WSSA) was used; it included 12 cups, a timer and a mat (Speed Stacks^®^, Speed Stacks Deutschland e.K.). The sport stacking cups (Speed Stacks^®^) had a width of 7.5 cm, a height of 9.5 cm and were green in color. The blue-colored grippy mat (StackMat Gen3^®^) was 23 cm × 75 cm in size and was placed on a wooden desk (see Figure 1). For non-invasive brain stimulation, a computer controlled HD-tDCS device with eight channels was used (Starstim, Neuroelectrics, Spain).

### HD-tDCS

**Figure 2:**
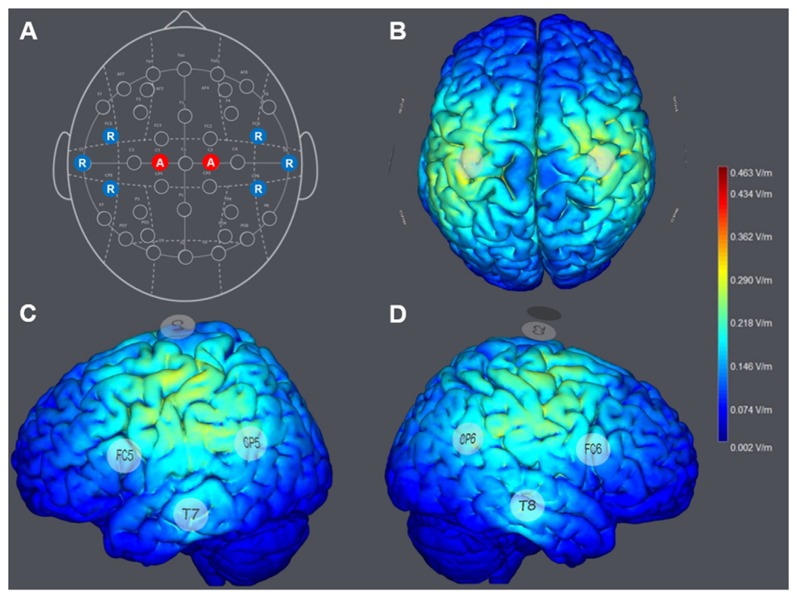
**(A)** Montage of the multichannel high-definition anodal transcranial direct current stimulation (HD-atDCS) according to the international 10-10-EEG system. Anodes (A) are colored in red and return electrodes (R; cathodes) are colored in blue. Computed general electric field (V/m) was generated by 1 milliampere (mA) anodal HD-tDCS to the left and right hemisphere, targeting the left and right primary motor cortices (M1; StimViewer, Neuroelectrics, Spain). **(B)** StimView above. **(C)** StimView left hemisphere**. (D)** StimView right hemisphere.

The configuration of HD-tDCS included eight electrodes positioned in a non-conductive neoprene cap. Direct current was applied using small, round gel electrodes (Ag/AgCl, 3.14 cm^2^), each filled with conductive (~4 S/m) electrolyte gel (Signa Gel, Parker Laboratories, Fairfield, NJ, USA). The electrode montage was based on the international 10-10-EEG system (Jurcak et al., 2007). To optimize the spatial orientation of the electric field of the HD-atDCS, all electrode positions were selected according to a computational model (Miranda et al., 2013; Ruffini et al., 2014), as shown in Figures 2B–D.

For the STIM-M1 group, anodal direct current was delivered at an intensity of 1 mA (current density 0.319 mA/cm^2^) to each stimulation electrode placed at C1 and C2, which covered the arm and hand areas of the left and right M1. Six return electrodes were placed at FC5, T7, CP5, FC6, T8 and CP6 (Figure 2A). The return current was approximately evenly distributed to each return electrode (16.67%), resulting in a computational intensity of −0.333 mA (current density −0.105 mA/cm^2^) at each return electrode. Minimal cathodal effects induced at the return positions were expected to be negligible in terms of motor performance on the task. The applied multi-channel montage resulted in a computational electric field power of 0.463 V/m (see Figures 2B–D).

For the control group (SHAM), the same montage was used and sham HD-tDCS was applied, with only 30 s of real anodal stimulation that returned promptly to zero. The duration of each stimulation session was 15 min, starting and closing with a 30-s ramp-up and ramp-down phase. The double-blind design was carried out by the password-protected double-blind mode of the control software (Neuroelectrics Instrument Controller v 1.4, Neuroelectrics, Spain) to ensure blindness of participants and the investigator to the stimulation condition being applied. Before and during application, the impedance of each electrode was checked to ensure that it was below 10 kΩ.

### Experimental Tasks

Sport stacking is a complex bimanual sequential sensorimotor task. The purpose of the task is to stack a given number of cups up and down as fast as possible without making a mistake (e.g., correct order of cups, no cups dropped). A stack is an assembly of cups that is stacked up and stacked down in a predefined formation beginning from left to right via the alternate use of both hands. The cups must be grasped and held on their sides and a competitor is only allowed to handle one stack (one assembly of cups) at a time to prevent parallel stacking. In the present study, we used two stacks, as defined by the official WSSA: the 3-6-3 and the 1-10-1 stacks (see Figures 1A,B). One sport stacking trial consisted of the completion of three stacks (3 × 3-6-3 or 3 × 1-10-1) in a row.

The 3-6-3 stack (Figure 1A) consists of 12 cups divided into three stacks and comprising three cups on the left, six cups in the center and three cups on the right. The 3-stacks are stacked up one cup after the other. The 6-stack is formed using the so-called 3-2-1 method, which consists of three cups that are grasped with the right hand, two cups grasped with the left hand and one cup left on the mat. The 1-10-1 stack (Figure 1B) consists of 12 cups stacked on top of each other. One cup must be placed on the left, 10 cups are stacked in the center and one cup is placed on the right. To form the 10-stack, the competitor follows the 5-4-1 method, which consists of five cups grasped with the right hand, four cups grasped with the left hand, and one cup left on the mat.

The stacking time (ST) for each sport stacking trial (three completed stacks in a row) was measured in seconds (s) by a timer with sensory touchpads (Speed Stacks^®^Gen3). Each participant started the measurement on their own by removing both hands from the touchpads (start) and finished by touching the pads with both hands when the trial was successfully finished (stop). The displayed ST was noted by the investigator and no further time measurements were made, e.g., with a handheld clock. Thus, motor performance was operationalized as movement speed assessed by movement time necessary to complete the stacking task.

### Experimental Procedure

**Figure 3:**
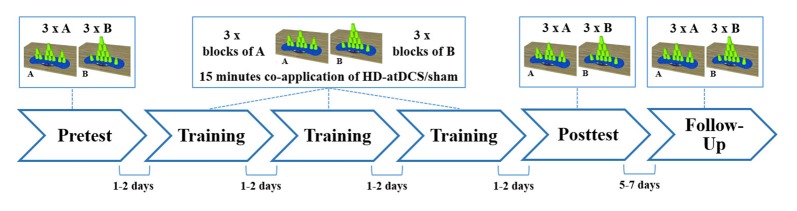
Schematic overview of the study design. All subjects participated at six experimental sessions. Measurement of sport stacking performance were obtained in a pre-, a post- and a follow-up test. Within three training sessions participants practiced sport stacking in alternated blocks (A = 3-6-3 and B = 1-10-1: ABABAB) beginning with block A and received concurrently either HD-atDCS or sham stimulation for 15 min. Each session took place at a separate day, with 1–2 days between each session, except for the follow-up test which was performed 5 to 7 days after the posttest.

Since practice is a major component in the induction of motor learning (Schmidt and Lee, 2011), a classic pre-posttest-design with follow-up test was chosen. Between the pre- and the posttest, all participants practiced sport stacking in three consecutive training sessions separated by 1–2 days (training period) in order to learn the two sport stacking formations. After the training period, motor performance was measured in a posttest to evaluate cumulative performance gains from day 1 to day 5. To evaluate consolidation effects, the participants were invited to perform both sport stacking tasks again after a retention interval of 5–7 days (Figure 3).

In the first session (pretest), the participants received instructions and a demonstration of the sport stacking task. For standardization purposes, all participants received the same written and verbal instructions and demonstration. During a 5-min acquisition phase, the participants could familiarize themselves with the sport stacking equipment and task. The investigator also controlled (additionally instructed) for correct execution. After the acquisition phase, a pretest was conducted that started with three 3-6-3 stack trials followed by three 1-10-1 stack trials.

According to the training protocol used in a previous study by our group (Pixa et al., 2017), training took place in the second, third and fourth sessions (see Figure 3). The volunteers were instructed to practice one block of the 3-6-3 stack (Block A) and one block of the 1-10-1 stack (Block B). Each block consisted of three stacking trials (3 × A or 3 × B). In each training session, three blocks of each stacking formation were alternately practiced beginning with block A (ABABAB). Thus, a total of nine trials of each stack were practiced per training session and a total number of 27 training trials for each type were performed after the completed training period. Each training session lasted 15 min, during which HD-atDCS/sham was simultaneously applied using the above-described stimulation parameters for an equal duration of 15 min. To prevent carryover effects from the stimulation, training sessions were separated by 1–2 days. Studies have indicated increased excitability of M1 from 30 min to 120 min when anodal tDCS is applied once, depending on stimulation duration and intensity (Kidgell et al., 2013; Kuo et al., 2013). Longer-lasting after-effects in excitability of up to 24 h were only found when tDCS was repeatedly applied within the same session (Bastani and Jaberzadeh, 2014). Therefore no cumulative stimulation effects were expected with our stimulation protocol since HD-atDCS was applied only once per session (day) concurrent with motor training and separated by 24–48 h. The posttest occurred in session five, and a follow-up test was carried out 5 to 7 days after the posttest (session 6).

Additionally, after the stimulation (verum/sham), the participants guessed whether they received a verum (yes) or a sham (no) stimulation. In the STIM-M1 group, 5 out of 16 participants correctly responded that they received verum stimulation. Nine out of 16 participants in the SHAM group correctly responded that they received sham stimulation, indicating that the response was based on chance.

### Statistical Analysis

Since the goal of sport stacking was to perform as fast as possible, and given its inherently competitive nature, the fastest ST (fST) on trials performed without any error were analyzed for pre-, post- and follow-up test performance. Kolmogorov-Smirnov tests indicated normal distribution of the data (*p* > 0.05). For a first global evaluation of motor performance improvements over the training period and between experimental groups, a three-way analysis of variance (ANOVA) with repeated measures (mixed-design) for TIME (PRE, POST and FOLLOW-UP), GROUP (STIM-M1 and SHAM) and FORMATION (3-6-3 and 1-10-1) was conducted. To gain a more differentiated picture of motor performance improvements, additional two-way ANOVAs with repeated measures (mixed-design) for TIME (PRE, POST and FOLLOW-UP) and GROUP (STIM-M1 and SHAM) were conducted once for each type of stacking. With regard to variability in motor abilities (He et al., 2016) and to address the possible influence of pretest scores on the outcome of treatments (Shmuelof and Krakauer, 2014), we conducted ANCOVAs that treated the pretest scores of 3-6-3 and 1-10-1 as covariates as recommended by Vickers and Altman (2001) and Morris (2008). Mauchly’s test was used to check for a violation of sphericity and Levene’s test was used to check for homogeneity. When a violation of sphericity was detected, Greenhouse-Geisser corrected *p*-values were reported. Effect sizes (Es) of ANOVAs and ANCOVAs were estimated as less biased partial omega-squares ($\omega_{\text{p}}^{\text{2}}$; Lakens, 2013), where $\omega_{\text{p}}^{\text{2}}$ > 0.01 indicates a small effect, $\omega_{\text{p}}^{\text{2}}$ > 0.06 a medium effect and $\omega_{\text{p}}^{\text{2}}$ > 0.14 a large effect (Cohen, 1988). To evaluate performance improvements for each type of stacking, two performance gain scores were calculated: (1) a Pre-Post Score was obtained by subtracting post-values from pre-values; and (2) a Pre-Follow-up Score was obtained by subtracting follow-up values from pre-values. Finally, differences in performance gains between the groups were evaluated by Bonferroni-corrected *t-tests* separately for both gain scores of each stack (3-6-3 and 1-10-1). Corrected Es of the *t*-tests were estimated according to the procedure provided by Morris (2008) as Cohen’s *d*, where *d* > 0.2 indicates a small effect, *d* > 0.5 indicates a moderate effect and *d* > 0.8 indicates a large effect (Cohen, 1988). The achieved power 1-*β* of the *t*-tests was computed *post hoc* (G*Power 3.1.9.2). The overall significance level was set at *p* ≤ 0.05. As recommended (e.g., by Fritz et al., 2012; Lakens, 2013), confidence intervals (CI) were reported for Es. Statistical calculations were done using Microsoft Excel 2013, IBM SPSS 23, and G*Power 3.1.9.2.

## Results

**Figure 4:**
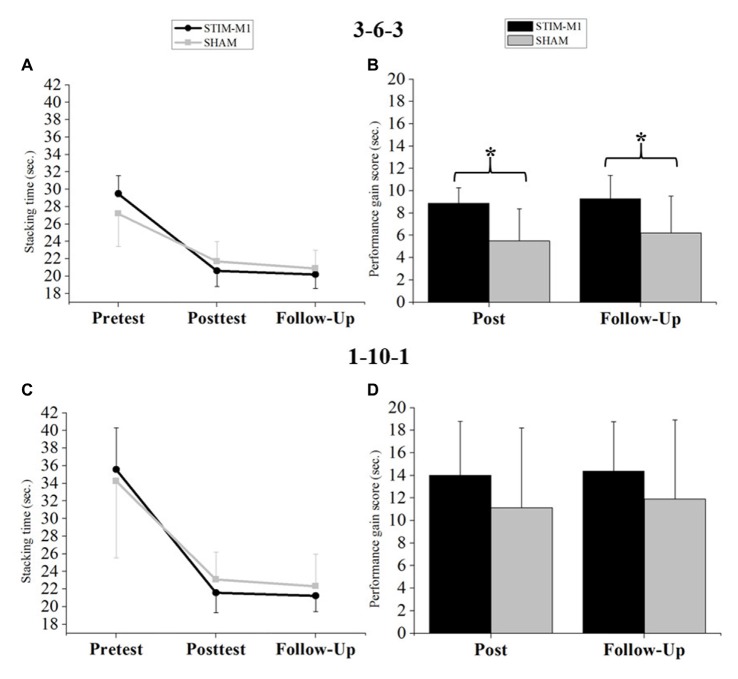
**(A)** Analysis of variance (ANOVA; mixed design) for the 3-6-3 stack. **(B)** Differences in performance gain scores (s) between both groups in the 3-6-3 stack. **(C)** ANOVA (mixed design) for the 1-10-1 stack. **(D)** Differences in performance gain scores (s) between both groups in the 1-10-1 stack. Error bars indicating standard deviation (SD) and *p < 0.05.

Statistical analysis of sport stacking performance (fST) using three-way ANOVA with repeated measures showed a significant main effect for TIME (*F*_(2,60)_ = 221.27, *p* < 0.001, $\omega_{\text{p}}^{\text{2}}$ = 0.874, 90% CI (0.827, 0.905)) and a significant TIME × GROUP interaction (*F*_(2,60)_ = 4.66, *p* < 0.05, $\omega_{\text{p}}^{\text{2}}$ = 0.104, 90% CI (0.017, 0.253)). Neither the interaction of FORMATION × GROUP (*F*_(2,60)_ = 0.53, *p* > 0.05, $\omega_{\text{p}}^{\text{2}}$ = 0.104, 90% CI (0.017, 0.253)) nor the three-way interaction among TIME × GROUP × FORMATION was significant. Finally, no significant main effect for GROUP was found (*p* > 0.05). To analyze the sport stacking performance for the 3-6-3 stack and the 1-10-1 stack, separate two-way ANOVAs with repeated measures were calculated. The two-way ANOVA with repeated measures for the 3-6-3 stack revealed a significant main effect of TIME (*F*_(2,60)_ = 239.87, *p* < 0.001, $\omega_{\text{p}}^{\text{2}}$ = 0.883, 90% CI (0.840, 0.912)) and a significant TIME × GROUP interaction (*F*_(2,60)_ = 11.21, *p* < 0.001, $\omega_{\text{p}}^{\text{2}}$ = 0.245, 90% CI (0.109, 0.396)), with improved performance in the STIM-M1 group. No significant main effect for GROUP was found (*p* > 0.05; Figure 4A). Bonferroni-corrected *post hoc t*-tests revealed no significant differences between both groups at any time point (*p* > 0.05). An ANCOVA for the 3-6-3 stack (fST) revealed that the covariate (pretest score 3-6-3) was significantly related to performance in the posttest (*F*_(1,29)_ = 26.81, *p* < 0.001, $\omega_{\text{p}}^{\text{2}}$ = 0.454, 90% CI (0.243, 0.621)). The significant effect of the stimulation on posttest performance remained after controlling for the effect of the pretest score (*F*_(1,29)_ = 13.36, *p* < 0.001, $\omega_{\text{p}}^{\text{2}}$ = 0.285, 90% CI (0.095, 0.489)). The pretest score was also significantly related to performance in the follow-up test (*F*_(1,29)_ = 8.73, *p* < 0.05, $\omega_{\text{p}}^{\text{2}}$ = 0.200, 90% CI (0.042, 0.416)) and the stimulation had still a significant effect on follow-up test performance (*F*_(1,29)_ = 4.80, *p* < 0.05, $\omega_{\text{p}}^{\text{2}}$ = 0.109, 90% CI (0.005, 0.327)). Analysis of performance improvements for the 3-6-3 stack by Bonferroni-corrected *t-tests* revealed significant differences between groups in both performance gain scores: (1) Pre-Post Score (*t*_(30)_ = −4.25, *p* < 0.05, *d* = −1.503, 95% CI (−2.282, −0.703)), with an achieved power of 1-*β* = 0.99. (2) Pre-Follow-up Score: (*t*_(30)_ = −3.14, *p* < 0.05, *d* = −1.110, 95% CI (−1.849, −0.355)), with an achieved power of 1-*β* = 0.92. Both performance gain scores indicate stronger improvements for the STIM-M1 group (Figure 4B).

Analysis of sport stacking performance (fST) for the 1-10-1 stack using two-way ANOVA with repeated measures (pre-, post- and follow-up test) showed a significant main effect of TIME (*F*_(2,60)_ = 142.21, *p* < 0.001, $\omega_{\text{p}}^{\text{2}}$ = 0.818, 90% CI (0.751, 0.862)). However, no significant TIME × GROUP interaction emerged (*p* > 0.05). Again, no significant main effect for GROUP was found (*p* > 0.05; Figure 4C) and Bonferroni-corrected *post hoc t*-tests showed no significant differences (*p* > 0.05). The ANCOVA for the 1-10-1 stack (fST) revealed that the covariate (pretest score 1-10-1) was significantly related to performance in the posttest (*F*_(1,29)_ = 11.41, *p* < 0.05, $\omega_{\text{p}}^{\text{2}}$ = 0.251, 90% CI (0.072, 0.461)). The significant effect of stimulation on posttest performance remained after controlling for the effect of the pretest score (*F*_(1,29)_ = 4.77, *p* < 0.05, $\omega_{\text{p}}^{\text{2}}$ = 0.108, 90% CI (0.046, 0.327)). The pretest score was also significantly related to performance in the follow-up test (*F*_(1,29)_ = 14.80, *p* < 0.001, $\omega_{\text{p}}^{\text{2}}$ = 0.308, 90% CI (0.112, 0.508)) but no significant stimulation effect was found on follow-up test performance (*F*_(1,29)_ = 2.80, *p* = 0.105, $\omega_{\text{p}}^{\text{2}}$ = 0.055, 90% CI (0.000, 0.265)). For the 1-10-1 stack, the performance improvements showed no significant differences between groups (*p* > 0.05; Figure 4D).

## Discussion

The central purpose of this study was to examine the effects of HD-atDCS applied over the left and right M1 on the performance of the sequential sensorimotor task of sport stacking compared to sham stimulation. Overall, the results demonstrate that the application of HD-atDCS concurrently with motor training over multiple days significantly improved cumulative performance in sport stacking (fST) in the stimulation group (STIM-M1). This effect remained after a 1-week retention interval, reflected by superior performance gain scores in the follow-up test in STIM-M1. This finding fits well with our hypothesis that anodal stimulation of both M1s leads to performance gains on a bimanual task compared to sham stimulation, and is in line with previous reports on cumulative improvements (Reis et al., 2009, 2015; Gomes-Osman and Field-Fote, 2013; Saucedo Marquez et al., 2013; Pixa et al., 2017). In a differentiated analysis, however, a clear and significant positive effect with sufficient effect sizes was only found for the 3-6-3 task, and not for the 1-10-1 task, although a similar pattern was observed that did not reach statistical significance. This result is only partially in line with our hypothesis. In spite of the randomization of group assignment, the pretest values for the 3-6-3 stack varied slightly between the groups, which might have had an impact on the outcome score and may have biased the treatment effects of HD-atDCS. However, the ANCOVAs that treated pretest scores as covariates showed significant correlations to posttest scores. There was still a significant and strong effect of the stimulation, which means that the significant interaction was not only attributable to baseline performance differences but also considerably to the stimulation itself. Generally, our results support previous studies reporting positive tDCS effects on unimanual motor behavior (for a review, see Buch et al., 2017) and extend these findings to bimanual motor performance (Gomes-Osman and Field-Fote, 2013; Pixa et al., 2017).

Given the fact that M1 is a major source of motor output and is substantially involved in motor consolidation, our data reveal that training with concomitant stimulation influences the motor processes of bimanual movements. In our analysis, both groups showed improved performance from pre- to posttest, demonstrating that training alone, as well as training combined with HD-atDCS, resulted in cumulative learning from day 1 to day 5. However, the performance gain in the posttest was significantly higher for STIM-M1 than for SHAM; the STIM-M1 group had a lower performance level at pretest and ended with a higher performance level in the posttest. This higher magnitude performance gain for STIM-M1 may be attributable to increased excitability in bilateral M1 (Reis and Fritsch, 2011; Stagg et al., 2011) and indicates enhanced motor learning due to neuroplastic changes in the motor system associated with LTP (Reis et al., 2009; Dayan and Cohen, 2011; Reis and Fritsch, 2011). However, it must be noted that only the performance gain on the 3-6-3 stack was significantly higher for STIM-M1 (not the 1-10-1 stack).

The interpretation that increased excitability in M1 led to superior performance gains in the STIM-M1 group is supported by studies that have reported increased bilateral activation in M1 during bimanual movements (McCombe Waller et al., 2008; Byblow et al., 2012; Neva et al., 2012). Thus, bilateral HD-atDCS combined with bimanual training may have boosted the cortical excitability in both M1 compared to bimanual training alone. Furthermore, sport stacking requires the alternate (asynchronous) use of both hands, and, as was briefly reviewed in the introduction, asynchronous bimanual movements elicit stronger bilateral activation in both M1 compared to synchronous bimanual movements (Haslinger et al., 2004). This may also have contributed to superior performance when HD-atDCS was co-applied with the asynchronous task of sport staking. It remains to be seen whether these effects vanish when participants perform sport stacking in a synchronized pattern, with synchronous movements of both hands. Although the comparison of synchronous vs. asynchronous bimanual movements would be an interesting question, a) this was out of scope of the present approach, and b) parallel stacking (i.e., synchronous movements) is expressly forbidden (according to the regulations of the WSSA).

One may argue that the interaction in absolute performance originated in higher baseline values (i.e., lower task performance) in the STIM-M1 group. Nevertheless, superior performance gains for STIM-M1 were preserved at the follow-up test after a 1-week retention interval. Consequently, and in line with recent findings (Pixa et al., 2017), these results indicate that our HD-atDCS protocol was sufficient to influence motor consolidation of the stacking task, possibly due to the well-described excitability increase induced by anodal tDCS (Nitsche and Paulus, 2000; Nitsche et al., 2005) combined with the excitatory effect of bimanual motor training (McCombe Waller et al., 2008; Neva et al., 2012). This effect might have led to stronger neuroplastic changes (increased LTP) in the motor cortex compared to pure motor training alone or stimulation alone without simultaneous motor activity (Reis and Fritsch, 2011; Stagg et al., 2011). It may also explain why no retention effect was found by Gomes-Osman and Field-Fote (2013). Moreover, the present study used a similar study design and stimulation protocol as was used in a previous study by our group (Pixa et al., 2017), which revealed comparable results with cumulative effects over the training period as well as after a retention interval. In that study, we observed improved dexterity in unimanual as well as in bimanual tasks (PPT) after 3 days of training. This supports our assumption that bimanual skills are sensitive to bilateral HD-atDCS co-applied with motor skill training.

Unexpectedly, the acquisition of tasks were slightly differentially affected by stimulation, since the two-way ANOVAs showed a significant interaction for 3-6-3 but not for 1-10-1. Performance outcomes of the ANCOVAs, however, still revealed a significant effect of the stimulation after controlling for pretest values, with the exception of the follow-up test performance for the 1-10-1 stack. These findings indicate that pretest performance might be an important factor (He et al., 2016) to prospectively take into account for training and stimulation protocols (Shmuelof and Krakauer, 2014). Besides the higher ANCOVA effect sizes for the 3-6-3 compared to the 1-10-1 stack, the differential results might be explained by different demands on fine motor skills (e.g., dexterity) and cognitive workload between the tasks: both stacks differ not only in terms of the required motor sequence (3-6-3 vs. 1-10-1), the amount of repetition (the 6-stack must also be built within the 10-stack, and as such, the number of repetitions is doubled), and the refined fine motor skills needed to handle 3, 6 and 3 cups with both hands (maximum of 3 cups in the right hand and 2 cups in the left) as compared to 1, 10 and 1 cups (maximum of 5 cups in the right hand and 4 cups in the left), but also in terms of the cognitive work load of planning the 1-10-1 stack, which is higher or more complex than for the 3-6-3 stack. Thus, the 1-10-1 stack may require higher involvement of fine dexterity when handling more cups in both hands compared to the 3-6-3. Besides these motor aspects, handling more cups at the same time might also require additional cognitive resources to plan and build the formation: In 1-10-1, the 10-stack consists of ten cups built on four levels, with four cups forming the base, followed by three cups, two cups and one cup on top. In contrast, in the 3-6-3 stack, fewer cups are handled at the same time to build the formation of six cups on three levels, where three cups form the base, followed by two cups, and one cup on top (see Figure 2). Whether the stacking formations differ in terms of cognitive demand was not tested in the present study and requires further exploration. That slight differences in task complexity may had led to different demands and different performance outcomes is speculative, although it has already been reported that small task variations can lead to different performance outcomes—an effect that has been linked to the involvement of different learning processes, e.g., implicit learning (Nitsche et al., 2003; Saucedo Marquez et al., 2013; Coffman et al., 2014). Thus, whether or not the higher demand for precise fine-motor skills and cognitive processes in the 1-10-1 task requires a longer training period and/or a varied stimulation protocol warrants further investigation.

Alternatively, our method of alternating training might have caused interference effects known to occur when two explicit motor tasks are trained in an alternating fashion (Brashers-Krug et al., 1996; Krakauer et al., 2005; Krakauer and Shadmehr, 2006). However, whether possible interference effects differentially influenced the sensitivity to neural excitation induced by HD-atDCS is highly speculative and appears to be unlikely, since both tasks yielded a similar performance pattern. Therefore, differences in the fine-motor skills required, repetition and complexity are more likely to account for our findings and should be explored in future studies in order to develop appropriate training and stimulation protocols for clinical and rehabilitative use.

### Limitations

The present study underlines and extends findings of tDCS-induced behavioral effects on bimanual motor skills. We focused our attention on bimanual motor performance in an experimental group receiving HD-atDCS compared to a sham control group. No further stimulation conditions in terms of an active control group (e.g., receiving tDCS with other parameters or on another brain area) were included. Because of the strongly expected learning effects in sport stacking, no within-subject design could be carried out, but future studies should consider such a design, e.g., in regard to inter-subject variability of tDCS effects (Wiethoff et al., 2014). Furthermore, no online tDCS effects on motor performance and learning were examined; this should be done in future studies to gain deeper insights into the direct effects of tDCS on motor performance. Since we used a complex bimanual sensorimotor task, it remains to be seen whether comparable effects will also be found in other more standardized bimanual motor tasks (e.g., a bimanual reaching and/or grasping task). Lastly, in the present study, no direct measurement of neurophysiological processes was done. Thus, we can only speculate about the neural mechanisms underlying the behavioral effects. In the present approach, the stimulation of left and right M1 might have influenced transcallosal pathways, considering that anodal stimulation of M1 has an excitatory effect on the ipsilateral M1 but a small inhibitory effect on the contralateral M1 (Vines et al., 2008; Stagg et al., 2009). To the best of our knowledge, no data is available regarding the effect of simultaneous anodal tDCS of both M1s and its effects on transcallosal communication (e.g., IHI). This was out of the scope of the present study and should be investigated in future studies.

## Conclusion

Following on previous studies that stimulated M1 by tDCS and showed successful modulation of unimanual motor performance, our findings indicate that HD-atDCS bilaterally applied to both M1s elicits improved bimanual motor performance. Although significant effects were clearly found for one of our tasks (3-6-3 stack) and only partially for the other (1-10-1 stack), strong effect sizes ($\omega_{\text{p}}^{\text{2}}$) and high statistical power (1-*β*) indicate that the present findings represent a valid starting point to fill a relevant gap in the tDCS literature concerning stimulation effects on complex bimanual movements. The findings are important for basic research as well as for different populations, such as patients suffering from neurological diseases and their associated bilateral motor impairments, and may help in the development of treatments to enhance motor recovery of the upper limbs.

## Author Contributions

NHP contributed to the conceptualization and realization of the study, performed the data acquisition, analysis and interpretation, wrote the manuscript and acted as corresponding author. FS substantially contributed to the data analysis and interpretation and wrote parts of the manuscript. MD contributed to the conceptualization and design of the study, and was involved in the data analysis and interpretation. He critically revised the manuscript and approved the final version and its content.

## Conflict of Interest Statement

The authors declare that the research was conducted in the absence of any commercial or financial relationships that could be construed as a potential conflict of interest.

## References

[B1] Alam M. Truong D. Q. Khadka N. Bikson M. (2016). Spatial and polarity precision of concentric high-definition transcranial direct current stimulation (HD-tDCS). Phys. Med. Biol. 61, 4506–4521. 10.1088/0031-9155/61/12/4506 27223853

[B2] Bastani A. Jaberzadeh S. (2012). Does anodal transcranial direct current stimulation enhance excitability of the motor cortex and motor function in healthy individuals and subjects with stroke: a systematic review and meta-analysis. Clin. Neurophysiol. 123, 644–657. 10.1016/j.clinph.2011.08.029 21978654

[B3] Bastani A. Jaberzadeh S. (2014). Within-session repeated a-tDCS: the effects of repetition rate and inter-stimulus interval on corticospinal excitability and motor performance. Clin. Neurophysiol. 125, 1809–1818. 10.1016/j.clinph.2014.01.010 24582469

[B4] Brashers-Krug T. Shadmehr R. Bizzi E. (1996). Consolidation in human motor memory. Nature 382, 252–255. 10.1038/382252a0 8717039

[B5] Brunoni A. R. Nitsche M. A. Bolognini N. Bikson M. Wagner T. Merabet L.. (2012). Clinical research with transcranial direct current stimulation (tDCS): challenges and future directions. Brain Stimul. 5, 175–195. 10.1016/j.brs.2011.03.002 22037126 PMC3270156

[B6] Buch E. R. Santarnecchi E. Antal A. Born J. Celnik P. A. Classen J.. (2017). Effects of tDCS on motor learning and memory formation: a consensus and critical position paper. Clin. Neurophysiol. 128, 589–603. 10.1016/j.clinph.2017.01.004 28231477

[B7] Byblow W. D. Stinear C. M. Smith M.-C. Bjerre L. Flaskager B. K. McCambridge A. B. (2012). Mirror symmetric bimanual movement priming can increase corticomotor excitability and enhance motor learning. PLoS One 7:e33882. 10.1371/journal.pone.0033882 22457799 PMC3310871

[B8] Carter M. J. Maslovat D. Carlsen A. N. (2015). Anodal transcranial direct current stimulation applied over the supplementary motor area delays spontaneous antiphase-to-in-phase transitions. J. Neurophysiol. 113, 780–785. 10.1152/jn.00662.2014 25376785

[B9] Carter M. J. Maslovat D. Carlsen A. N. (2017). Intentional switches between coordination patterns are faster following anodal-tDCS applied over the supplementary motor area. Brain Stimul. 10, 162–164. 10.1016/j.brs.2016.11.002 27838274

[B10] Cauraugh J. H. Lodha N. Naik S. K. Summers J. J. (2010). Bilateral movement training and stroke motor recovery progress: a structured review and meta-analysis. Hum. Mov. Sci. 29, 853–870. 10.1016/j.humov.2009.09.004 19926154 PMC2889142

[B11] Coffman B. A. Clark V. P. Parasuraman R. (2014). Battery powered thought: enhancement of attention, learning and memory in healthy adults using transcranial direct current stimulation. Neuroimage 85, 895–908. 10.1016/j.neuroimage.2013.07.083 23933040

[B12] Cohen J. (1988). Statistical Power Analysis for the Behavioral Sciences. Hillsdale, NJ: Erlbaum.

[B14] Dayan E. Censor N. Buch E. R. Sandrini M. Cohen L. G. (2013). Noninvasive brain stimulation: from physiology to network dynamics and back. Nat. Neurosci. 16, 838–844. 10.1038/nn.3422 23799477 PMC4876726

[B13] Dayan E. Cohen L. G. (2011). Neuroplasticity subserving motor skill learning. Neuron 72, 443–454. 10.1016/j.neuron.2011.10.008 22078504 PMC3217208

[B15] Doppelmayr M. Pixa N. H. Steinberg F. (2016). Cerebellar, but not motor or parietal, high-density anodal transcranial direct current stimulation facilitates motor adaptation. J. Int. Neuropsychol. Soc. 22, 928–936. 10.1017/s1355617716000345 27152869

[B16] Doyon J. Benali H. (2005). Reorganization and plasticity in the adult brain during learning of motor skills. Curr. Opin. Neurobiol. 15, 161–167. 10.1016/j.conb.2005.03.004 15831397

[B17] Edwards D. Cortes M. Datta A. Minhas P. Wassermann E. M. Bikson M. (2013). Physiological and modeling evidence for focal transcranial electrical brain stimulation in humans: a basis for high-definition tDCS. Neuroimage 74, 266–275. 10.1016/j.neuroimage.2013.01.042 23370061 PMC4359173

[B18] Foerster R. M. Carbone E. Koesling H. Schneider W. X. (2011). Saccadic eye movements in a high-speed bimanual stacking task: changes of attentional control during learning and automatization. J. Vis. 11:9. 10.1167/11.7.9 21665985

[B19] Foerster R. M. Carbone E. Koesling H. Schneider W. X. (2012). Saccadic eye movements in the dark while performing an automatized sequential high-speed sensorimotor task. J. Vis. 12:8. 10.1167/12.2.8 22323821

[B20] Fritz C. O. Morris P. E. Richler J. J. (2012). Effect size estimates: current use, calculations and interpretation. J. Exp. Psychol. Gen. 141, 2–18. 10.1037/a0024338 21823805

[B21] Gomes-Osman J. Field-Fote E. C. (2013). Bihemispheric anodal corticomotor stimulation using transcranial direct current stimulation improves bimanual typing task performance. J. Mot. Behav. 45, 361–367. 10.1080/00222895.2013.808604 23796102

[B22] Grefkes C. Eickhoff S. B. Nowak D. A. Dafotakis M. Fink G. R. (2008). Dynamic intra- and interhemispheric interactions during unilateral and bilateral hand movements assessed with fMRI and DCM. Neuroimage 41, 1382–1394. 10.1016/j.neuroimage.2008.03.048 18486490

[B23] Halsband U. Lange R. K. (2006). Motor learning in man: a review of functional and clinical studies. J. Physiol. Paris 99, 414–424. 10.1016/j.jphysparis.2006.03.007 16730432

[B24] Hardwick R. M. Rottschy C. Miall R. C. Eickhoff S. B. (2013). A quantitative meta-analysis and review of motor learning in the human brain. Neuroimage 67, 283–297. 10.1016/j.neuroimage.2012.11.020 23194819 PMC3555187

[B25] Haslinger B. Erhard P. Altenmüller E. Hennenlotter A. Schwaiger M. Gräfin von Einsiedel H.. (2004). Reduced recruitment of motor association areas during bimanual coordination in concert pianists. Hum. Brain Mapp. 22, 206–215. 10.1002/hbm.20028 15195287 PMC6871883

[B26] He K. Liang Y. Abdollahi F. Fisher Bittmann M. Kording K. Wei K. (2016). The statistical determinants of the speed of motor learning. PLoS Comput. Biol. 12:e1005023. 10.1371/journal.pcbi.1005023 27606808 PMC5015831

[B27] Jurcak V. Tsuzuki D. Dan I. (2007). 10/20, 10/10 and 10/5 systems revisited: their validity as relative head-surface-based positioning systems. Neuroimage 34, 1600–1611. 10.1016/j.neuroimage.2006.09.024 17207640

[B28] Kidgell D. J. Goodwill A. M. Frazer A. K. Daly R. M. (2013). Induction of cortical plasticity and improved motor performance following unilateral and bilateral transcranial direct current stimulation of the primary motor cortex. BMC Neurosci. 14:64. 10.1186/1471-2202-14-64 23815634 PMC3701480

[B29] Kilbreath S. L. Heard R. C. (2005). Frequency of hand use in healthy older persons. Aust. J. Physiother. 51, 119–122. 10.1016/s0004-9514(05)70040-4 15924514

[B31] Krakauer J. W. Ghez C. Ghilardi M. F. (2005). Adaptation to visuomotor transformations: consolidation, interference, and forgetting. J. Neurosci. 25, 473–478. 10.1523/jneurosci.4218-04.2005 15647491 PMC6725486

[B30] Krakauer J. W. Shadmehr R. (2006). Consolidation of motor memory. Trends Neurosci. 29, 58–64. 10.1016/j.tins.2005.10.003 16290273 PMC2553888

[B32] Kuo H.-I. Bikson M. Datta A. Minhas P. Paulus W. Kuo M.-F.. (2013). Comparing cortical plasticity induced by conventional and high-definition 4 × 1 ring tDCS: a neurophysiological study. Brain Stimul. 6, 644–648. 10.1016/j.brs.2012.09.010 23149292

[B33] Lakens D. (2013). Calculating and reporting effect sizes to facilitate cumulative science: a practical primer for *t*-tests and ANOVAs. Front. Psychol. 4:863. 10.3389/fpsyg.2013.00863 24324449 PMC3840331

[B34] Liebetanz D. Nitsche M. A. Tergau F. Paulus W. (2002). Pharmacological approach to the mechanisms of transcranial DC-stimulation-induced after-effects of human motor cortex excitability. Brain 125, 2238–2247. 10.1093/brain/awf238 12244081

[B35] McCombe Waller S. Forrester L. Villagra F. Whitall J. (2008). Intracortical inhibition and facilitation with unilateral dominant, unilateral nondominant and bilateral movement tasks in left- and right-handed adults. J. Neurol. Sci. 269, 96–104. 10.1016/j.jns.2007.12.033 18336839 PMC2910578

[B36] McKune A. J. Mortimer J. Custard S. Krysztofiak J. (2011). Effect of sport stacking on motor and cognitive function in grade three learners. Med. Sci. Sport Exer. 43:256 10.1249/01.mss.0000400702.38011.a2

[B37] Miranda P. C. (2013). Physics of effects of transcranial brain stimulation. Handb. Clin. Neurol. 116, 353–366. 10.1016/b978-0-444-53497-2.00029-2 24112908

[B38] Miranda P. C. Mekonnen A. Salvador R. Ruffini G. (2013). The electric field in the cortex during transcranial current stimulation. Neuroimage 70, 48–58. 10.1016/j.neuroimage.2012.12.034 23274187

[B39] Morris S. B. (2008). Estimating effect sizes from pretest-posttest-control group designs. Organ. Res. Methods 11, 364–386. 10.1177/1094428106291059

[B40] Neva J. L. Legon W. Staines W. R. (2012). Primary motor cortex excitability is modulated with bimanual training. Neurosci. Lett. 514, 147–151. 10.1016/j.neulet.2012.02.075 22405809

[B41] Nikolin S. Loo C. K. Bai S. Dokos S. Martin D. M. (2015). Focalised stimulation using high definition transcranial direct current stimulation (HD-tDCS) to investigate declarative verbal learning and memory functioning. Neuroimage 117, 11–19. 10.1016/j.neuroimage.2015.05.019 25987365

[B42] Nitsche M. A. Paulus W. (2000). Excitability changes induced in the human motor cortex by weak transcranial direct current stimulation. J. Physiol. 527, 633–639. 10.1111/j.1469-7793.2000.t01-1-00633.x 10990547 PMC2270099

[B43] Nitsche M. A. Schauenburg A. Lang N. Liebetanz D. Exner C. Paulus W.. (2003). Facilitation of implicit motor learning by weak transcranial direct current stimulation of the primary motor cortex in the human. J. Cogn. Neurosci. 15, 619–626. 10.1162/089892903321662994 12803972

[B44] Nitsche M. A. Seeber A. Frommann K. Klein C. C. Rochford C. Nitsche M. S.. (2005). Modulating parameters of excitability during and after transcranial direct current stimulation of the human motor cortex. J. Physiol. 568, 291–303. 10.1113/jphysiol.2005.092429 16002441 PMC1474757

[B45] Oldfield R. C. (1971). The assessment and analysis of handedness: the edinburgh inventory. Neuropsychologia 9, 97–113. 10.1016/0028-3932(71)90067-4 5146491

[B46] Pixa N. H. Steinberg F. Doppelmayr M. (2017). High-definition transcranial direct current stimulation to both primary motor cortices improves unimanual and bimanual dexterity. Neurosci. Lett. 643, 84–88. 10.1016/j.neulet.2017.02.033 28229937

[B47] Priori A. (2003). Brain polarization in humans: a reappraisal of an old tool for prolonged non-invasive modulation of brain excitability. Clin. Neurophysiol. 114, 589–595. 10.1016/s1388-2457(02)00437-6 12686266

[B48] Reis J. Fritsch B. (2011). Modulation of motor performance and motor learning by transcranial direct current stimulation. Curr. Opin. Neurol. 24, 590–596. 10.1097/WCO.0b013e32834c3db0 21968548

[B49] Reis J. Fischer J. T. Prichard G. Weiller C. Cohen L. G. Fritsch B. (2015). Time- but not sleep-dependent consolidation of tDCS-enhanced visuomotor skills. Cereb. Cortex 25, 109–117. 10.1093/cercor/bht208 23960213 PMC4415064

[B50] Reis J. Schambra H. M. Cohen L. G. Buch E. R. Fritsch B. Zarahn E.. (2009). Noninvasive cortical stimulation enhances motor skill acquisition over multiple days through an effect on consolidation. Proc. Natl. Acad. Sci. U S A 106, 1590–1595. 10.1073/pnas.0805413106 19164589 PMC2635787

[B51] Ruffini G. Fox M. D. Ripolles O. Miranda P. C. Pascual-Leone A. (2014). Optimization of multifocal transcranial current stimulation for weighted cortical pattern targeting from realistic modeling of electric fields. Neuroimage 89, 216–225. 10.1016/j.neuroimage.2013.12.002 24345389 PMC3944133

[B52] Saucedo Marquez C. M. Zhang X. Swinnen S. P. Meesen R. Wenderoth N. (2013). Task-specific effect of transcranial direct current stimulation on motor learning. Front. Hum. Neurosci. 7:333. 10.3389/fnhum.2013.00333 23847505 PMC3696911

[B53] Schambra H. M. Abe M. Luckenbaugh D. A. Reis J. Krakauer J. W. Cohen L. G. (2011). Probing for hemispheric specialization for motor skill learning: a transcranial direct current stimulation study. J. Neurophysiol. 106, 652–661. 10.1152/jn.00210.2011 21613597 PMC3154830

[B54] Schmidt R. A. Lee T. D. (2011). Motor Control and Learning: A Behavioral Emphasis. Champaign ILL: Human Kinetics.

[B55] Shmuelof L. Krakauer J. W. (2014). Recent insights into perceptual and motor skill learning. Front. Hum. Neurosci. 8:683. 10.3389/fnhum.2014.00683 25232311 PMC4153040

[B57] Stagg C. J. Jayaram G. Pastor D. Kincses Z. T. Matthews P. M. Johansen-Berg H. (2011). Polarity and timing-dependent effects of transcranial direct current stimulation in explicit motor learning. Neuropsychologia 49, 800–804. 10.1016/j.neuropsychologia.2011.02.009 21335013 PMC3083512

[B56] Stagg C. J. Nitsche M. A. (2011). Physiological basis of transcranial direct current stimulation. Neuroscientist 17, 37–53. 10.1177/1073858410386614 21343407

[B58] Stagg C. J. O’Shea J. Kincses Z. T. Woolrich M. Matthews P. M. Johansen-Berg H. (2009). Modulation of movement-associated cortical activation by transcranial direct current stimulation. Eur. J. Neurosci. 30, 1412–1423. 10.1111/j.1460-9568.2009.06937.x 19788568

[B59] Swinnen S. P. Wenderoth N. (2004). Two hands, one brain: cognitive neuroscience of bimanual skill. Trends Cogn. Sci. 8, 18–25. 10.1016/j.tics.2003.10.017 14697399

[B60] Udermann B. E. Mayer J. M. Murray S. R. Sagendorf K. (2004). Influence of cup stacking on hand-eye coordination and reaction time of second-grade students 1. Percept. Motor. Skill 98, 409–414. 10.2466/pms.98.2.409-414 15141904

[B61] Vancleef K. Meesen R. Swinnen S. P. Fujiyama H. (2016). tDCS over left M1 or DLPFC does not improve learning of a bimanual coordination task. Neuroreport 6:35739. 10.1038/srep35739 27779192 PMC5078840

[B62] Vickers A. J. Altman D. G. (2001). Statistics notes: analysing controlled trials with baseline and follow up measurements. BMJ 323, 1123–1124. 10.1136/bmj.323.7321.1123 11701584 PMC1121605

[B63] Villamar M. F. Volz M. S. Bikson M. Datta A. Dasilva A. F. Fregni F. (2013). Technique and considerations in the use of 4x1 ring high-definition transcranial direct current stimulation (HD-tDCS). J. Vis. Exp. 77:e50309. 10.3791/50309 23893039 PMC3735368

[B64] Vines B. W. Cerruti C. Schlaug G. (2008). Dual-hemisphere tDCS facilitates greater improvements for healthy subjects′ non-dominant hand compared to uni-hemisphere stimulation. BMC Neurosci. 9:103. 10.1186/1471-2202-9-103 18957075 PMC2584652

[B65] Waters-Metenier S. Husain M. Wiestler T. Diedrichsen J. (2014). Bihemispheric transcranial direct current stimulation enhances effector-independent representations of motor synergy and sequence learning. J. Neurosci. 34, 1037–1050. 10.1523/JNEUROSCI.2282-13.2014 24431461 PMC3891947

[B66] Wessel M. J. Zimerman M. Timmermann J. E. Heise K. F. Gerloff C. Hummel F. C. (2016). Enhancing consolidation of a new temporal motor skill by cerebellar noninvasive stimulation. Cereb. Cortex 26, 1660–1667. 10.1093/cercor/bhu335 25604611

[B67] Wiethoff S. Hamada M. Rothwell J. C. (2014). Variability in response to transcranial direct current stimulation of the motor cortex. Brain Stimul. 7, 468–475. 10.1016/j.brs.2014.02.003 24630848

